# B Cells in the Multiple Sclerosis Central Nervous System: Trafficking and Contribution to CNS-Compartmentalized Inflammation

**DOI:** 10.3389/fimmu.2015.00636

**Published:** 2015-12-24

**Authors:** Laure Michel, Hanane Touil, Natalia B. Pikor, Jennifer L. Gommerman, Alexandre Prat, Amit Bar-Or

**Affiliations:** ^1^Département de Neurosciences, Centre de Recherche du Centre Hospitalier de l’Université de Montréal, Montréal, QC, Canada; ^2^Neuroimmunology Unit, Montreal Neurological Institute, McGill University, Montréal, QC, Canada; ^3^Department of Immunology, University of Toronto, Toronto, ON, Canada; ^4^Experimental Therapeutics Program, Montreal Neurological Institute, McGill University, Montréal, QC, Canada

**Keywords:** B cells, multiple sclerosis, central nervous system, meningeal inflammation, trafficking

## Abstract

Clinical trial results of peripheral B cell depletion indicate abnormal proinflammatory B cell properties, and particularly antibody-independent functions, contribute to relapsing MS disease activity. However, potential roles of B cells in progressive forms of disease continue to be debated. Prior work indicates that presence of B cells is fostered within the inflamed MS central nervous system (CNS) environment, and that B cell-rich immune cell collections may be present within the meninges of patients. A potential association is reported between such meningeal immune cell collections and the subpial pattern of cortical injury that is now considered important in progressive disease. Elucidating the characteristics of B cells that populate the MS CNS, how they traffic into the CNS and how they may contribute to progressive forms of the disease has become of considerable interest. Here, we will review characteristics of human B cells identified within distinct CNS subcompartments of patients with MS, including the cerebrospinal fluid, parenchymal lesions, and meninges, as well as the relationship between B cell populations identified in these subcompartments and the periphery. We will further describe the different barriers of the CNS and the possible mechanisms of migration of B cells across these barriers. Finally, we will consider the range of human B cell responses (including potential for antibody production, cytokine secretion, and antigen presentation) that may contribute to propagating inflammation and injury cascades thought to underlie MS progression.

## Introduction

Roles of B cells in central nervous system (CNS) inflammatory diseases have been investigated in patients and through elegant animal model studies. Here, we will focus on studies carried out in human, with animal work described in more detail elsewhere in this issue. B cell responses have long since been recognized in MS with variable degrees of evidence implicating them in both early/relapsing and later/progressive disease (Table 1). The abnormal presence of antibodies in the CNS continues to represent the most consistent immunodiagnostic feature in patients with MS. Cerebrospinal fluid (CSF)-restricted oligoclonal bands (OCBs) are reported in the CSF of over 90% of MS patients throughout disease stages (1–5). Over the years, pathological implication of B cells has included the demonstration of antimyelin antibodies inside phagocytic cells within MS lesions (6, 7), the observation that the most common demyelinating lesion pattern (Pattern II) is characterized by prominent deposition of antibodies and complement (8), and the more recent descriptions of meningeal immune cell collections that can be B cell rich (9–12). The latter were first described in a proportion of patients with progressive forms of MS and subsequently also within meninges of patients considerably earlier in their disease course (9–12). Molecular analyses of the Immunoglobulin (Ig) variable gene region of B cells and plasma cells from active parenchymal lesions, the CSF, or meninges of MS patients have revealed the persistence of (presumably antigen driven) clonotypes that are shared between these three different CNS subcompartments (10, 13–18). Antibodies generated from clonally expanded plasma cells derived from the CSF of MS patients were capable of both binding human and mouse CNS tissue, and causing complement-mediated demyelination and astrocyte activation in spinal cord explants (19). In spite of the long-standing implication of clonally expanded B cell populations and abnormal antibodies in the MS CNS, the antigens recognized by these antibodies are still subject of debate and different targets have been suggested such as viruses, axoglial proteins, and glycolipids (20–25). The more recent work-deriving antibodies from CSF-expanded B cell clones of MS patients suggest that they may preferentially target neurons and astrocytes (19, 26). The significance of serum antibodies to molecules, such as MOG and KIR4.1, also continues to be investigated (27–33).

**Table 1:** **Strength of evidence implicating B cells in early/relapsing and later/progressive MS**.

	Early/relapsing MS	Later/progressive MS
Clinical arguments	Anti-CD20 therapy robustly limits new focal inflammatory brain lesions and MS relapses (34–36)	Anti-CD20 therapy may limit worsening of disability in (*post hoc*) subgroup analysis of PPMS patients (younger patients; those exhibiting gadolinium enhancing lesions) (37)
	PLEX may improve resolution of steroid refractory relapses (38)	
Biological arguments	CSF OCB already present early in relapsing MS course in many patients; IgG levels (39) and presence of IgM OCBs have been associated with MS activity (39–41)	CSF OCB present in majority of patients later in MS course; some implication that their presence is associated with more aggressive or progressive course (39, 40)
	Abnormal autoantibodies against MOG (27–30) and KIR4.1 (31, 32) reported in some patients with MS; clinical significance remains unclear IgG transfer from MS patients can induce complement-mediated demyelination in animals (27, 42)	
	Dynamic exchange of B cell clones found in MS CNS and periphery, and evidence that activation/maturation may occur in the periphery (13, 43)	Shared B cell/PC clones within different CNS subcompartments including parenchymal lesions, CSF as well as meninges (10)
Pathological arguments	Common lesion type in pathologic classification of demyelinating lesions notable for deposition of immunoglobulin (Ig) and complement (8) Antibodies and myelin fragments have been identified within phagocytic cells in MS lesions (6, 44)	
	Meningeal inflammation including presence of B cells, as well as subpial cortical demyelinating lesions can be features of early MS (9)	B cell-rich meningeal aggregates associated with subpial cortical lesions reported as more common in progressive forms of MS (10–12)

*PLEX, plasma exchange; OCB, oligoclonal bands; MOG, myelin oligodendrocyte glycoprotein; PC, plasma cell*.

**Figure 1:**
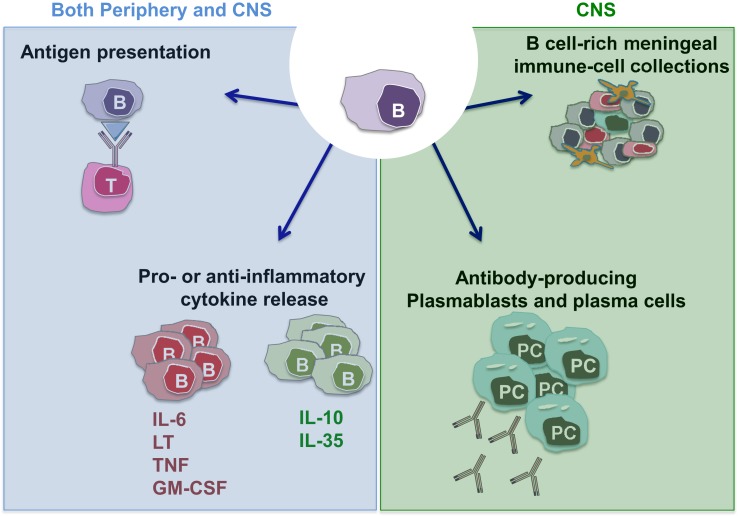
**Potential MS-relevant B cell responses**. (1) B cells can function as efficient antigen-presenting cells (APC) especially in context of cognate B cell:T cell interactions. This may activate pathogenic T cells that in turn contribute to disease propagation. (2) B cells and/or plasma cells have the potential to produce anti-inflammatory cytokines (such as IL-10 and IL-35) but also proinflammatory cytokines (IL-6, LTα, TNFα, and GM-CSF). A lack of balance involving over-propensity of B cells to produce proinflammatory cytokines and their deficient production of anti-inflammatory cytokines has been demonstrated in patients with MS. Such B cell responses within the CNS may contribute to propagating CNS-compartmentalized inflammation. (3) B cells can differentiate into plasmablasts and plasma cells, which can elaborate pathogenic autoantibodies (and possibly also cytokines). (4) Similar to their established roles in normal lymphoid architecture formation, B cells may release factors that contribute to the formation and/or maintenance of persisting immune cell aggregates in the meninges of MS patients.

The observation that B cell depletion with anti-CD20 monoclonal antibodies substantially limits new relapsing MS disease activity (34–36, 45, 46) has made it clear that B cells play important roles in the immune cascades underlying CNS inflammation and has reinvigorated research efforts to elucidate mechanisms underlying such B cell roles. Of interest in this regard, is the observation that while anti-CD20 therapy rapidly reduces new relapsing MS disease activity, the abnormalities in CSF antibody measures seem to persist in the face of the therapeutic benefit (47). This indicates that the therapeutic mechanisms of action by which B cell depletion limits new MS relapses reflect at least in part antibody-independent roles of B cells. Indeed, B cells are now recognized to have multiple functions that may contribute to MS pathogenesis, in addition to their capacity to differentiate into antibody-secreting cells (plasmablasts/plasma cells) (Figure 1). B cells can be highly efficient antigen-presenting cells (APC) to T cells when presenting antigens that they initially recognize with their surface B cell receptor (BCR) (48). In this context, Harp et al. reported that memory B cells in MS patients can efficiently present neuro-antigens to T cells (49, 50). Moreover, activated B cells can modulate the local inflammatory response of both T cells and myeloid cells through secretion of proinflammatory or anti-inflammatory cytokines (described in detail in Li et al., in this issue). Some B cells support proinflammatory functions of other cells through production of TNFα, IL-6, GM-CSF, and Lymphotoxin-alpha (51–55), while IL-10 and IL-35 producing B cells possess anti-inflammatory (regulatory) roles (53, 56–58). In MS, B cells seem to be abnormally polarized toward a more proinflammatory phenotype (54, 55, 59, 60), and defects in their regulatory function have also been suggested by some but not all authors (55, 59–62).

Anti-CD20 therapy has also been studied in Phase III trials of patients with primary progressive MS (PPMS). Treatment in the initial trial using rituximab, failed to limit disease progression though a benefit was suggested in the subgroup of younger patients, and those with evidence of focal inflammatory brain lesions (37). The follow-up ORATORIO study of anti-CD20 (ocrelizumab) focused on younger patients who were closer to clinical PPMS disease-onset, and demonstrated a modest treatment benefit in limiting the rate of progression of neurological disability (63, 64). A number of factors may limit a more robust effect of peripheral B cell depletion on progressive MS biology. Meningeal immune cell aggregates in which B cells can be a prominent feature may not be as efficiently targeted by anti-CD20 antibodies that only weakly penetrate the CNS. It is also possible that long-lived plasma cells (that do not express CD20) and the antibodies they generate may play a more important role in progressive forms of MS compared to relapsing MS. B cells are known to play important roles in the formation of normal lymphoid follicle architecture (65, 66). Observations of B cell-rich immune cell collections in the meninges of MS patients [some of which recapitulate lymphoid follicle-like features (9, 11, 12) and reviewed by Pikor and Gommerman, in this issue] raise the intriguing possibility that B cells contribute to the formation and/or maintenance of such structures. In doing so, the B cells may contribute to propagation of inflammation within the MS CNS. Some or all of the diverse functions of B cells which are now thought to contribute to inflammatory responses in the periphery of MS patients may also be relevant within the CNS.

It now appears likely that functionally distinct B cells contribute to the MS disease process through diverse mechanisms within the distinct disease compartments and throughout different stages of the disease. Peripheral proinflammatory B cells play an important role in relapsing disease mechanisms (see Li et al., in this issue), whereas meningeal collections of B cells potentially participate in the maintenance and propagation of CNS-compartmentalized disease. This review will focus on studies that implicate human B cells within the CNS of MS patients. We will highlight available findings form human studies that (I) consider the sites and characteristics of B cells within the MS CNS subcompartments, including CSF, parenchyma, and meninges; (II) how B cells might get there (barriers/trafficking); and (III) what they might do there (responses that may be relevant to CNS injury processes).

### Where Are B Cells Within the MS CNS?

**Figure 2:**
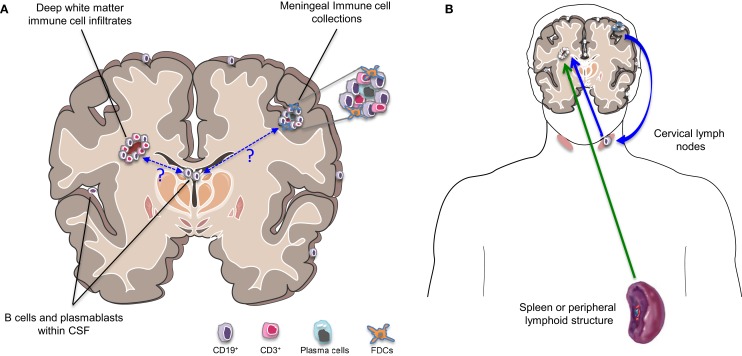
**B cells in different compartments and implication for MS disease activity**. **(A)** Cells of the B cell lineage (including primarily memory B cells, plasmablasts, and plasma cells) are found to persist in the inflamed MS CNS and occupy multiple subcompartments. These include the cerebrospinal fluid (CSF), parenchymal white matter lesions, and collections of immune cells within the meninges, which can be B cell rich. Somatic mutation analysis has demonstrated that the same B cell clones may occupy all three CNS subcompartments. Exactly how and where such clones initially access the CNS and how they communicate across these CNS subcompartments remains largely unknown. **(B)** The traditional view has held that new MS disease activity is triggered by activation of immune cells in the periphery (possibly triggered by pathogen-associated molecules recognized by cross-reactive T cells; referred to as molecular mimicry) and subsequent trafficking of the activated cells into the CNS (green arrow). However, the demonstration that the CNS clearly has lymphatics that drain into cervical lymph nodes and evidence from somatic mutation analysis indicating bidirectional trafficking of B cells between the CNS and the periphery (with much of the activation and clonal expansion apparently occurring in the periphery) suggests that relapses may also be “invited” from within the CNS (blue arrows). This might occur if proinflammatory B cells exit the chronically inflamed CNS carrying CNS antigens, which they may then present to T cells in the draining cervical lymph nodes with subsequent trafficking of the activated T cells into the CNS.

B cells, plasmablasts, and/or plasma cells have been described in several subcompartments of the CNS of patients with MS, including the CSF, parenchyma, and meninges (Figure 2A). Emerging studies are adding to our understanding of the profiles of such cells as well as the relationship between such cells in the different CNS compartments.

#### Cerebrospinal Fluid B Cells in MS

Early studies investigating CSF cytology suggested that the number and the relationship of B cells to other CSF immune cells (principally monocytes) may be associated with MS disease severity and progression (67). In particular, a high ratio (predominance of B cells) was associated with more rapid disease progression, whereas a low ratio (predominance of monocytes) was found in patients with slower progression (67). Subsequent work demonstrated that CSF B cells in MS CSF are largely class-switched IgD^−^IgM^−^ memory B cells (68) and that the main B cell effector subset are short-lived plasmablasts (69, 70). Over the years, several groups carried out somatic hypermutation analysis of the variable region of the heavy chain immunoglobulin (Ig V_H_) in B cells obtained from CSF of MS patients in comparison to circulating blood B cells obtained from the patients at the same time (14–18). A consistent finding was that MS CSF harbored increased frequencies of clonally expanded B cells (with post-germinal center memory characteristics) compared to the blood. The mutations appeared to be highly concentrated within the CDR3 region, which has been taken to indicate an antigen-driven selection process of B cells accumulating in the CSF of patients. Presorting and amplifying the variable region of the IgG gene from both total CD19^+^ B cells and CD138^+^ plasma cell/plasmablasts purified from the CSF of MS patients revealed that both sorted subsets harbored somatically mutated expanded clones (71). The repertoire within the CD138^+^ subset was more restricted though little sequence overlap was observed between the CD19^+^ and CD138^+^ repertoires (71). More recently, analysis of genes for IgM-chains in CSF B cells of MS patients revealed extensive accumulation of somatic hypermutation and clonal expansion in IgM-producing B cells (72). Whether or not these cells initially trafficked into the CNS as naïve B cells, their coexpression of activation-induced cytidine deaminase (AID, an enzyme crucial for somatic hypermutation and class switch recombination of antibodies, that is normally expressed during activation of B cells in germinal centers) provides further support that the intrathecal milieu in patients with MS sustains accumulation of germinal center-like experienced B cells that can produce both IgM and IgG antibodies. Additional elegant work examining both CSF B cell IgG-H and Ig-κ chains transcriptomes, as well as the oligoclonal Ig proteomes derived from the same CSF of patients with relapsing-remitting MS, showed a correspondence between CSF Ig proteomes and B cell Ig transcriptomes, providing the most direct evidence that expanded CSF B cell clones are responsible for producing the abnormal Ig that comprises the CSF OCB in MS patients (73).

#### Parenchymal B Cells in MS Brains

Most histopathologic studies of MS are based on autopsy tissues, which tend to be obtained relatively late in the disease course. When patients undergo biopsy earlier in the course of disease, such tissues may not be representative of typical MS pathology as biopsies tend to be done only when the lesion and/or clinical presentation are sufficiently atypical. Thus, available insights into the distribution and magnitude of B cell infiltrates within the MS CNS (whether in the parenchyma or meninges) largely reflect longer-standing disease states. With respect to the parenchyma, studies have generally indicated that the classical deep white matter perivascular demyelinating lesions of MS typically exhibit relatively few B cells and plasma cells compared to the greater abundance of myeloid cells and T cells (8, 74, 75). At the same time, demyelinating lesion classification has identified “Type II” lesions (that exhibit considerable Ig and complement deposition) as the most common demyelinating lesion type in MS (8, 74). It is noteworthy that in a study of 26 active lesions from 11 patients diagnosed with relatively early MS, demyelinated lesions reportedly exhibited considerable numbers of B cells as well as IgG-positive plasma cells, in addition to T cells and myeloid cells (75, 76). This raises the possibility that B cells and plasma cells may be a more common feature of early, as compared to later, MS parenchymal lesions. Somatic mutation analysis of B cells and plasma cells isolated from both parenchymal lesions and CSF of the same MS patients at autopsy demonstrated clonally expanded and somatically hypermutated populations within the tissue samples, as well as shared clones populating the tissue and CSF (77). Shared clones were subsequently demonstrated in parenchyma and meninges of the patients (10). These observations point to relatedness of expanded B cell and plasma cell clones in both parenchymal and extraparenchymal subcompartments of the MS CNS, at least later in disease.

#### B Cells Within Meningeal Immune Cell Collections in MS

Cellular immune aggregates have been reported in the meninges of patients with MS, some of which were found to be rich in B cells, and have been referred to as “ectopic follicles” or “follicle-like structures” based on their resemblance to tertiary lymphoid tissues (TLT) (11, 12). Early studies described these structures mainly in subsets of relatively late-phase SPMS and PPMS patients (11, 12, 78, 79). Presence of these meningeal immune cell collections was associated with more aggressive clinical disease and a greater extent of tissue injury in the subjacent cortical regions. The pattern of the demyelinating subpial cortical injury associated with meningeal inflammation involved a gradient of microglial activation, reduced numbers of oligodendrocytes, and neuronal loss, such that the most severe injury was present in the most superficial cortical layers. Meningeal immune cell aggregates were most commonly found in the deep sulci of the temporal, cingulate, insula, and frontal cortex (79). Based on these initial reports, the prevailing concept was that meningeal inflammation in MS is a feature of a subset of patients with late/progressive disease. However, recent imaging studies demonstrate leptomeningeal contrast enhancement in the brain of individuals with RRMS (80) and substantial meningeal inflammation has also been described in biopsy material obtained from patients relatively early in their MS disease course (9). While these biopsies were obtained for diagnostic purposes of atypical deep white matter lesions, the biopsy trajectories captured meningeal and cortical tissue that exhibited the typical features of cortical MS injury, and demonstrated considerable meningeal inflammation. The cortical tissue underlying the meningeal immune cell collections in these early cases depicted a similar demyelinating injury pattern (with enhanced microglial activation, reduced number of oligodendrocytes, and neuronal/neuritic loss) as was described in the more chronic cases (11, 78). Further characterization of meningeal immune cell aggregates in MS indicated that at least some are enriched in proliferating (Ki67^+^) CD20^+^ B cells. Presence of some plasma cells/plasmablasts, CD8^+^ and CD4^+^ T cells, and CXCL13-producing CD35^+^ cells with follicular dendritic cell characteristics were also described (12, 79, 81). As noted, somatic hypermutation analysis of the BCR in B cells and plasma cells isolated from meningeal immune cell aggregates, as well as parenchymal lesions and CSF from the same MS patients, demonstrated that related B cell clones populate all three compartments (10), again underscoring the relatedness of clonally expanded B cells found in the MS CNS. CNS-infiltrating B cells are also clonally related to peripheral B cells (13, 14, 18) raising intriguing questions about the dynamics involved in trafficking of B cells into the CNS and among these distinct CNS subcompartments as discussed below. It is still not clear whether formation of meningeal immune cell collections occurs commonly and throughout the different (early and late) phases of disease, and whether their presence contributes to, or is merely the consequence of underlying tissue injury. Indeed, some groups did not identify the presence of meningeal immune cell aggregates or a relation between such aggregates and cortical injury (82). These discrepancies may reflect true biological heterogeneity across patients, transient presence of meningeal inflammation (for example, during periods of more active CNS inflammation), or technical reasons as these structures tend to be very small (≤100 μm in thickness) and may be lost depending on the approach to tissue processing.

### What Routes Might B Cells Use to Infiltrate the CNS?

#### Anatomical Routes to Cross the Blood/CNS Barriers

The CNS (comprising the brain and spinal cord) which was historically referred to as immune privileged is now referred to as immune specialized with the understanding that peripherally derived immune cells do patrol the CNS as part of normal physiologic immune surveillance (83–85). This immune specialization is conferred by the presence of barriers that restrict the passage of large molecules and limit broader cell infiltration. While the most known of these specialized barriers is the blood–brain barrier (BBB), two less well studied but nonetheless important other barriers are the blood–meningeal barrier (BMB) and the blood CSF barrier (BCB).

##### The Blood–Brain Barrier

The BBB is a structure formed by specialized endothelial cells (ECs) that separates the CNS from systemic circulation. CNS blood vessels are made of two main cell types: the ECs themselves and the mural cells that sit on the abluminal surface of the EC layer (i.e., pericytes and astrocytes). CNS ECs are characterized by the presence of tight junctions that limit the paracellular flux of solutes and by a very low rate of transcytosis. These properties permit a tight control of the exchange between the brain and the blood (86). Maintenance of the BBB is governed by both cellular and non-cellular elements that interact with the ECs. Astrocytes, pericytes, and the extracellular matrix together provide structural and functional support to the BBB (87–89). The term “neurovascular unit” (NVU) additionally refers to neurons and microglia cells that also contribute to this barrier. At the level of the postcapillary venule, two distinct basement membranes (endothelial and parenchymal) define the inner and outer border of the perivascular space. Basement membranes keep members of the NVU in place and regulate their intercellular cross-talk.

##### The Blood–Meningeal Barrier

The meninges are composed of three layers that surround the CNS (Dura mater, Arachnoid mater, and Pia mater) and contain the CSF located within the subarachnoid space. In the brain, the gray matter is directly adjacent to the meninges. While the meninges were initially considered as a mere physical barrier preventing entry of infections and toxins into the CNS, more recent findings have established this tissue as a site of active immunity in both health and disease (84, 85, 90). Similar to barrier membranes in the gut and lungs, the meninges can house a wide variety of immune-competent cells such as macrophages, dendritic cells, mast cells, innate lymphoid cells, and fibroblasts that can provide effective protection against microbes (91–93). As is the case with the other immunologically competent barriers, the meninges can also be the site of chronic inflammation in pathologic states. The description of meningeal immune cell collections associated with MS has reinforced the concept that the BMB could be an important pathway in immune cell CNS trafficking. Indeed, live imaging studies in animals demonstrate that lymphocytes cross the BMB prior to onset of CNS inflammation and appear to become reactivated in the subarachnoid space as part of disease instigation (94, 95). Relatively, little is known about the properties of ECs located in the meninges, which appear to differ in important ways from parenchymal ECs associated with the BBB. For example, meningeal microvessels lack the rich astrocytic ensheathment, which characterizes the microvessels in the CNS parenchyma (96).

##### The Blood CSF Barrier

Another port of entry into the CNS is the choroid plexus (CP) forming a barrier between the blood and CSF. This villous structure extends into the ventricular organs and is also responsible for producing the CSF. The CP is made up of a layer of epithelial cells surrounding a core of fenestrated capillaries and connective tissue, allowing the free diffusion of solutes from the blood toward the parenchyma through inter-endothelial gaps. The monolayer of epithelial cells has tight gap junctions that prevent the flux of macromolecules and cells and acts as a blood:CSF barrier (97). It has been shown that ICAM-1 and VCAM-1 are constitutively expressed by CP epithelial cells (98), and this barrier may be a port of entry of pathogenic TH17 cells during the commonly used animal model of CNS inflammation, experimental autoimmune encephalomyelitis (EAE), an influx mediated at least in part *via* CCR6/CCL20 interactions (99).

#### Molecular Mechanisms Underlying Cell Trafficking into the CNS

##### The Multistep Process of Leukocyte Extravasation

In healthy individuals, there is a very low rate of ongoing immune surveillance of the CNS. Immune cell migration across barriers is normally tightly regulated and involves a multistep process. These different steps include rolling, firm adhesion, crawling, and extravasation (97, 100–104). The initial contact between leukocytes and the endothelium is usually mediated by adhesion molecules of the selectin family. This first step allows the reduction of the leukocyte velocity in the bloodstream, hence allowing them to detect the chemokine factors secreted by, or bound to ECs. The binding of chemokines to their cognate receptors expressed on the surface of leukocytes leads to an increased avidity/affinity of interaction between cellular adhesion molecules (immunoglobulin family members such as VCAM1, ICAM1, ALCAM, and MCAM) and adhesion molecule receptors such as those of the integrin family, which contributes to firm adhesion of the cells to the endothelium. Subsequent leukocyte polarization and crawling (typically against the direction of blood flow) to sites permissive for diapedesis, requires the expression of ICAM1 and 2 (but not VCAM1) by ECs and is a prerequisite for immune cell diapedesis across the BBB (94).

Leukocytes can then migrate through inter-endothelial regions (diapedesis) or directly through the ECs themselves. Expression of several of these adhesion molecules has been found to be highly increased in MS tissue and is thought to contribute to the extravasation of leukocytes into the CNS parenchyma of patients (100–106). Different preferential pathways and molecular mechanisms of trafficking across the BBB have already been identified for T cells and monocytes [for review, see Ref. (97)]. Less is known concerning B cell migration into the CNS.

##### Molecules Implicated in B Cell Migration into the CNS

Natalizumab, which binds VLA-4, is one of the most potent therapies in RRMS. Studies have mainly focused on its impact on T cells migration across the BBB, but B cells express also high levels of VLA-4 (107, 108). A major role of VLA-4 in B cells migration across human adult brain-derived ECs has been shown *in vitro*, with a prominent role also identified for ICAM-1 (108). A recent study has reported that the selective inhibition of VLA-4 expression on B cells reduces the susceptibility to EAE by decreasing B cell accumulation inside the CNS but also by interfering with TH17/macrophage recruitment (109). Finally, another adhesion molecule named ALCAM (activated leukocyte cell adhesion molecule) seems to promote B cell trafficking into the CNS across the BBB (103). Nonetheless, little is known about whether distinct B cell subsets that have been implicated in MS utilize particular molecular pathways to get across the BBB, and whether and how B cells traffic across the other CNS barriers (BMB and CP), are among key questions that have not yet been elucidated.

#### Dynamics of B Cell Infiltration into the MS CNS

Until recently, the documentation of clonally expanded B cells in the MS CNS including CSF, lesions, and meninges, has been taken as evidence that B cell clonal expansion is driven (by one or more unknown antigens) within the CNS of patients (10, 13–18). More recent evidence points to the potential for more dynamic, bidirectional exchange of B cells between the CNS and periphery (Figure 2B), including clonal expansion that occurs in both compartments (13, 14). Since the initial study implicating active diversification of B cells on both sides of the BBB (18), two additional complementary studies confirmed that particular B cells found outside the CNS (in both peripheral blood and draining cervical lymph nodes), share clonality with B cells populating the brain (13, 14) and exhibit evidence of presumably antigen-driven expansion on both sides of the BBB. In one of these studies, using paired CNS tissue and draining cervical lymph nodes from the same patient source, not only were shared B cell clones identified in the two compartments, but the founding clones and much of the subsequent maturation involved in the bidirectional exchange, appeared to take place in the cervical lymph nodes rather than the CNS (13, 14). This could provide a mechanism for “epitope spread,” a phenomenon well described in animal studies whereby the antigenic target of the CNS inflammatory attack shifts over time as injury exposes additional epitopes (110). Supporting a role for B cells in such “epitope spread” in patients with MS are observations from antigen array studies indicating that the circulating repertoire of serum anti-CNS antibodies appears to expand in children with MS, yet constrict in children with monophasic CNS inflammatory disease, over time (111).

It is now also apparent that the CNS is not as “immune privileged” as previously thought, with organized lymphatic draining that allows CNS antigens and potential APC to exit from the CNS including the meninges and to access the periphery (84, 85). Based on these observations, one can challenge the prevailing view that MS relapses are invariably triggered by some external stimulus (e.g., pathogen exposure) resulting in peripheral immune cell activation and trafficking into the CNS. Instead, cells capable of antigen presentation, such as B cells, may drain from the CNS into the draining lymph nodes, and present CNS antigens to T cells with subsequent T cell activation and trafficking involved in new relapsing disease activity.

### How Might B Cells Within the CNS Contribute to MS Disease Mechanisms?

While the capacity of B cells to mediate aberrant T cell activation in the periphery could explain the substantial contribution of B cells to relapsing MS biology (evidenced by robust relapse-reduction following B cell depletion with anti-CD20 therapy), whether and how B cells may also contribute to progressive (non-relapsing) disease remains to be elucidated. The biology underlying CNS injury in progressive MS is now thought to involve a combination of degeneration and ongoing inflammation that is compartmentalized within the CNS (112). Such compartmentalized inflammation involves astrocyte and microglial activation, though the molecular mechanisms driving such chronic activation remain largely unknown. Since B cells are recognized to persist in the chronically inflamed MS CNS (10, 13, 14, 16), and evidence has mounted that B cells of patients with MS exhibit abnormal proinflammatory response profiles (54, 55, 59, 60), it has been tempting to consider whether B cells chronically residing in the CNS may contribute to propagating local injury processes even independent of B cell roles in relapsing disease biology. This concept is reinforced by reports of meningeal immune cell infiltrates which can be rich in B cells and that have now been identified in both early and late (9, 11, 12, 79) stages of MS. One potential mechanism by which B cells could contribute to ongoing injury is through secretion of CNS-directed autoantibodies (5, 6, 8). As noted, somatic mutation analysis has indicated that clonally expanded B cells and plasma cells are shared between the different CNS subcompartments (CSF, parenchyma, and meninges) (10). Moreover, CSF-derived B cell clones can produce antibody that binds CNS cells (including neurons and astrocytes) and can be shown to cause complement-mediated injury to such structures in CNS explants (19, 26). Antibody-independent contributions (e.g., Figure 1) of B cells to propagating inflammation in the MS CNS should also be considered. The great majority of B cells identified in the MS CNS (regardless of subcompartment) appear to be preferentially memory rather than naïve B cells (14, 15), and it is now recognized that memory B cells of MS patients may have particular proinflammatory propensities including the capacity to express exaggerated levels of immune activating molecules and proinflammatory cytokines (55, 59). This may be particularly relevant when considering meningeal B cell-rich immune collections and the subpial cortical demyelinating injury, which is now thought to importantly contribute to progressive loss of neurological function in patients with MS. These subpial demyelinating lesions are notable for microglial activation, astrogliosis, and neuronal loss, and their location may be associated with regions subjacent to areas of meningeal immune cell collections (81). It is intriguing to speculate whether particular B cell subsets persisting within such immune cell collections may impact the underlying glial neural cells through the release of specific soluble factors. In turn, what factors within the inflamed CNS milieu may sustain B cells in that environment? Does CNS persistence of particular B cell clones relate to the antigenic specificity of the B cells? In the case of primary CNS lymphoma, there is some evidence that specific recognition by tumoral B cells of CNS antigens contribute to fostering local tumor survival and proliferation (113), and such a mechanism may also contribute to persistence of B cells in the MS CNS. A number of features of the inflamed MS CNS may support B cells unrelated to their antigenic specificity. These include soluble factors known to support B cell survival that are produced by activated astrocytes and microglia – such as BAFF, IL-6, IL-10, and IL-15 – all reportedly found at increased levels within the CSF of MS patients (114–116). Some of these factors (BAFF and IL-6) also support the survival of plasma cells. In the context of EAE, plasmablasts and plasma cells have been implicated in regulating neuroinflammation through their production of cytokines such as IL-10 and IL-35, although it is unclear if this is occurring exclusively in the periphery (lymph nodes) or also in the CNS itself (56, 117). Thus, identifying the particular B cell subsets that preferentially migrate into, and are then fostered within, the MS CNS, and elucidating how they may contribute to propagating local injury responses are of considerable interest for future studies.

### Perspectives

The success of anti-CD20 therapies has made it clear that B cells contribute substantially to the initiation of MS relapses. Growing evidence suggests this largely reflects non-antibody-dependent proinflammatory roles of B cells in the periphery, where they can aberrantly activate disease-relevant T cells, which in turn traffic to the CNS and mediate relapses. The inflamed MS CNS appears to foster persistence of B cells and plasma cells and the same clonally expanded populations can be found within different CNS subcompartments (CSF, parenchyma, and meninges). There is an early appreciation that multiple distinct barriers separate the CNS from the periphery, including the BBB, meningeal, and choroidal interfaces. Elegant studies now underscore the bidirectional trafficking of B cells between the CNS and the periphery and reveal that maturation of expanded clones that populate the CNS of patients may be peripherally rather than centrally driven. Despite key advances, little is known about B cell contributions to the chronic non-relapsing CNS-compartmentalized inflammation that may underlie progressive tissue injury and worsening of disability in MS. A number of observations make such contributions (through both antibody-dependent and antibody-independent mechanisms) plausible and worthy of further study. Key observations reviewed here include the known persistence of B cells in the inflamed MS CNS of patients; the demonstration that CSF-derived B cell clones isolated from MS patients can bind CNS (including neurons and astrocytes) and cause complement-mediated injury; the now recognized abnormal proinflammatory response propensity of MS B cells; potential cross-talk between B cells and activated CNS glial cells; and the reported association between B cell-containing meningeal immune cell infiltrates and presence of the subpial cortical injury increasingly thought to underlie progressive decline of functions in patients with progressive MS. Future work should aim to address key remaining questions (Box 1) thereby shedding light on which functionally distinct B cell subsets are present in the different anatomical subcompartments in the CNS, which molecular mechanisms and barriers are involved in their trafficking into those sites, what their antigenic specificities are, how are they fostered in the local environment, how they interact with glial and neural cells and ultimately how they contribute to disease propagation in the MS CNS as compared to the case of NMO (Box 2). These insights will hopefully help guide novel therapeutic options that may prove as useful for limiting progressive disease biology as peripheral B cell depletion has been for limiting relapses.

Box 1 **B cells in the MS CNS: what remains to be elucidated?**

What are preferential routes of migration of B cells into the CNS?

Which molecules are involved in migration of distinct B cell subsets?

What are the molecular mechanisms that favor B cell persistence in the MS CNS?

What are the antigenic specificities of abnormal CSF immunoglobulins (Ig) in MS?

Which Ig are disease-relevant vs. an epiphenomenon of chronic activation?

Which response profiles (proinflammatory/anti-inflammatory) characterize CNS B cells?

What are the different roles of B cells within immune cell aggregates in the meninges?

What are the interactions between B cells and other cells in the inflamed MS CNS?

- Do they have direct effects on oligodendrocytes and neurons?
- Do they present antigen/s to T cells? Do they modulate T cell activation/polarization?
- Do they influence astrocyte/microglia activation/polarization and *vice versa*?

Box 2 **The case of Neuromyelitis Optica (NMO).**

For years, neuromyelitis optica (NMO) was largely considered a variant of MS until the discovery of serum antibodies to the water channel aquaporin (AQP)-4, which distinguished patients with NMO from those with MS (118, 119). A growing range of clinical syndromes found to harbor such antibodies has since lead to the characterization of “NMO spectrum disorders” (NMOSD) as a pathophysiologic spectrum that should be considered distinct from multiple sclerosis (120). Unlike MS (in which no particular antibody has been firmly linked to pathophysiology), a convergence of pathologic (121–125) and clinical (126–131) observations supports a pathophysiologic role of anti-AQP-4 antibodies in NMOSD [reviewed by Ref. (132, 133)]. While anti-AQP-4 antibodies are thought to be pathogenic in NMOSD, the observation that decreased NMO relapses seen following anti-CD20-mediated B-cell depletion do not correlate well with changes in anti-AQP4 antibody titers (134–136), indicates that the role of B cells in NMO may extend beyond antibody production. Such antibody-independent roles may include the capacity of B cells activate T cells and/or myeloid cells, as also implicated in MS. The observation that anti-AQP-4 antibodies are more readily detectable in serum rather than CSF of NMOSD patients has raised the question whether pathogenic antibodies are exclusively generated in the periphery and subsequently access the CNS, or whether plasmablasts and plasma cells that secrete such antibodies can be induced and fostered within the CNS. A recent study indicates that during NMO exacerbations, a substantial fraction of the intrathecal Ig proteome is generated by B cells of both peripheral and central origin (137). This suggests that in order for NMO therapies aiming to target the source of anti-AQP-4 antibodies to be most effective, they will need to access both the periphery and the CNS. Pathologically, NMO is characterized by an astrocytopathy with vasculocentric deposition of complement, vascular fibrosis, and eosinophilic infiltration, with associated white matter and gray matter injury. Meningeal immune cell collections with follicle-like features and cortical demyelination do not appear to be features of NMO pathology (138).

## Author Contributions

LM, HT, and AB-O designed and wrote the manuscript. AP, JG, and NP helped critically the manuscript.

## Conflict of Interest Statement

Laure Michel, Hanane Touil, Natalia B. Pikor, Jennifer L. Gommerman, and Alexandre Prat have no conflicts of interest. Amit Bar-Or has participated as a speaker in meetings sponsored by and received consulting fees and/or grant support from Biogen Idec, Diogenix, Genentech, Sanofi-Genzyme, GlaxoSmithKline, Novartis, Ono Pharma, Teva Neuroscience, Receptos Inc., Roche, and Merck/EMD Serono.
